# Exercise-based telerehabilitation in chronic low back pain – a scoping review

**DOI:** 10.1186/s12891-024-07952-7

**Published:** 2024-11-23

**Authors:** Jenny Sivertsson, Ninni Sernert, Kristina Åhlund

**Affiliations:** 1 Institute of Clinical Science, Department of Orthopaedics, Sahlgrenska Academy, University of Gothenburg, Gothenburg, Sweden; 2 Department of Physiotherapy, NU Hospital Group, Uddevalla, Sweden; 3 Department of Research and Development, NU Hospital Group, Trollhättan, Sweden; 4 Department of Health Sciences, University West, Trollhättan, Sweden

**Keywords:** Telerehabilitation, Low back pain, Physiotherapy, Exercise therapy, Scoping review, SAMR model

## Abstract

**Background:**

Low back pain is a major global health problem. Physiotherapy involving exercises is considered first-line treatment. In recent years digital tools including telerehabilitation have increased, but the interventions are diverse. The aim of this study was to map how telerehabilitation approaches are used in studies evaluating exercise-based rehabilitation in patients with chronic low back pain.

**Methods:**

A systematic literature search was conducted in PubMed, Cinahl and Cochrane Central between January 2017 and January 2024 for original studies on adults, 18 years or older, with chronic low back pain who received exercise-based telerehabilitation.

**Results:**

The database search resulted in 1019 articles. Out of 37 full texts that were screened 28 articles were included in the analysis. The included studies showed a wide variation regarding technological solutions, interventions and outcome measures. The exercise-based telerehabilitation was usually delivered asynchronously via a smartphone application. The most common clinical outcome measure was pain and disability/physical function. Telerehabilitation compared to conventional exercise therapy showed similar clinical improvements.

**Conclusions:**

This scoping review confirms the heterogeneity within this research area but also contributes by mapping and demonstrating some knowledge gaps in the literature. Further research focusing on synchronous and group interventions are needed. The new technologies described in the included studies provide added value through functional improvements and task redesign.

**Trial registration:**

OSF https//doi.org/10.17605/OSF.IO/EMKCG.

## Background

Low back pain is a major global health problem. It is one of the most common causes of disability [1, 2] and leads to high costs for society [3]. Low back pain occurs in all age groups, most commonly among people of working age and the prevalence is higher in women than in men [2]. Most adults will suffer from low back pain at some point in their lives [1]. Cases of low back pain are usually short-lasting, but recurrence is common. Only a few people experience persistent pain [2].

Low back pain is defined as pain and discomfort localised in the lumbosacral region, with or without radiating leg pain [4] and is classified as chronic when it persists longer than 3 months, the time expected for tissue healing [5]. Most cases of low back pain are labelled as nonspecific, which is done when symptoms are not attributable to a specific pathology [2]. The condition is often described as complex and includes biological, psychological and social factors [2]. In the assessment and treatment of chronic low back pain, international guidelines are based on a biopsychosocial model [6] which emphasizes the importance of information about staying physically active and supports self-care strategies [7]. However, physiotherapy is also an important component in the management of low back pain, and individually tailored exercise therapy is recommended [7–9].

Exercise therapy is described as “a regimen or plan of physical activities designed and prescribed for specific therapeutic goals. Its purpose is to restore normal musculoskeletal function or to reduce pain caused by diseases or injuries” [10]. In the literature, exercise therapy is described as an “umbrella term” that includes different types of exercises, such as mobility, aerobic, stabilisation, stretching, balance, coordination and muscle strengthening exercises that can vary in intensity, frequency, and duration. Exercise therapy can be given as a single treatment or as part of a multidisciplinary treatment programme. It can be done individually or in groups, be supervised or consist of home exercises, be done on land or in water and include only one’s own body or specific equipment [11].

Conventional physiotherapy treatment of low back pain in Sweden take place in primary care and involves face-to-face clinical assessment, an individually tailored exercise programme and patient education including self-care strategies, with regular follow-ups by the physiotherapist [12]. Systematic reviews have evaluated the effect of different types of exercise therapy in chronic low back pain, showing that Pilates, strength, stabilisation and aerobic exercise often are effective in reducing pain and disability and therefore recommended [8, 9, 11, 13–16]. However, there is still a lack of evidence whether one specific type of exercise therapy is more effective than others.

Adherence to the exercise programme and the ability to perform the exercises correctly are important factors in enabling a good effect of the treatment. However, adherence over time is challenging, and to be compliant, patients with chronic low back pain often need support from healthcare providers, such as supervised exercise and the use of new technology to visualise feedback [17]. Other methods which are shown to contribute to increased adherence are the use of patient motivational strategies, including positive feedback, reinforcement of patients’ efforts, advice about self-reminders, use of an exercise diary and treatment contracts [18].

In recent years, especially in connection with the Covid-19 pandemic, the use of telecommunications technologies in healthcare has increased and continues to develop. In the literature, the terminology used regarding digital healthcare services is diverse, but commonly used terms are telemedicine, e-health, telehealth and telerehabilitation [19]. Telerehabilitation refers to the delivery of rehabilitation services via information and communication technologies and includes different types of interventions, such as exercise programmes, education and strategies for improved self-management [20]. Telerehabilitation involves different modes of delivery, and the communication can take place synchronously, that is, in real-time, or asynchronously, which entails a delay between the sending and receiving of the data. Different telecommunication technologies are used, for example web-based applications, platforms, text messages, video games and virtual reality [19, 21]. There are previous studies evaluating the effectiveness of telerehabilitation in patients with musculoskeletal conditions and chronic low back pain indicating results comparable to those of traditional, face-to-face interventions [22–26]. Studies have also highlighted good patient acceptability [27, 28].

However, it is important to consider the added value that is provided when traditional physiotherapy is replaced with digital solutions. The substitution, augmentation, modification, and redefinition framework (SAMR) [29] originates from the field of education and can serve as a guide in the creation and evaluation of new technological solutions of how digitalisation contributes to innovation and gains [30]. The framework consists of four hierarchical levels where the substitution level means a direct tool replacement and no functional change, the technology acts as a direct replacement for previous analogue working methods, while the augmentation level means some form of functional improvement in relation to the patient’s or physiotherapist’s experience or treatment goals, for example written instructions are supplemented with audio and video. The modification and redefinition levels involve a significant redesign or a new task that was previously inconceivable, for example the use of a platform as a shared contact surface [29]. Exercise-based physiotherapy for patients with low back pain involves, among other things, teaching the correct execution of specific exercises, as well as education in self-care and pain management. Based on both digitalisation and education, the framework should thus be able to contribute to structuring the results of the current study.

Physiotherapy involving exercise therapy is considered first-line treatment in patients with chronic low back pain [7]. In recent years digital tools have been introduced on a large scale in healthcare, including physiotherapy, where telerehabilitation has increased and continues to develop. However, scientific evaluations are still lacking, and among what has been presented so far, the variation is large in terms of terminology, interventions, ways of conveying digital exercise therapy and outcome measures. There is thus a great need for a structured evaluation of what is already known, to be able to develop the concept further, to the benefit of both patients and healthcare. The results may help and guide in clinical practice when considering telerehabilitation as an alternative to conventional treatment of chronic low back pain.

### Aim

To study how telerehabilitation approaches are used in studies evaluating exercise-based rehabilitation in patients with chronic low back pain.

### Questions

1. What technological methods are used within the concept of telerehabilitation in exercise-based interventions in patients with chronic low back pain?
2. How are the clinical outcomes evaluated?
3. What are the main results of the exercise-based telerehabilitation interventions?

## Methods

### Design

A scoping review according to the methodological framework of Arksey and O’Malley [31] with five major steps was conducted: (1) Identifying the research question, (2) Identifying relevant studies, (3) Selecting the studies, (4) Charting the data, (5) Collating, summarising and reporting the results. To guide the question development the PCC mnemonic (population, concept and context) was used [32]. The reporting process was guided using the Preferred Reporting Items for Systematic Reviews and Meta-Analyses extension for Scoping Reviews (PRISMA-ScR) [33]. The study protocol was pre-registered with Open Science Framework (OSF): 10.17605/OSF.IO/EMKCG.

Clinical trial number: not applicable.

### Search strategy and eligibility criteria

A systematic literature search was first conducted in January 2023 and then an additional search in January 2024 in the electronic databases, PubMed, Cinahl and Cochrane Central. The search strategy was first developed in PubMed and then optimised for the other databases. The search strategy followed the accepted three-step method for searching [34]. First, an initial limited search was done in a selection of relevant databases, followed by an analysis of the text words contained in the title and abstract, and of the index word used to describe the article. Then, a second search was performed using all identified keywords and index terms across all included databases. Lastly, the reference list of all identified articles was searched for additional studies.

The initial limited search used the following Boolean search string (telerehabilitation) AND (exercise OR “physical therapy” OR physiotherapy OR “exercise-based treatment”) AND (“low back pain”).

The second search used the following Boolean search string: (telerehabilitation OR telemedicine OR telehealth OR “tele health” OR “tele rehabilitation” OR eHealth OR videoconferenc* OR digital* OR mhealth OR mobile OR smartphone OR app OR virtual* OR remote OR video) AND (“low back pain” OR “chronic low back pain”) AND (exercise OR training OR “exercise-based rehabilitation” OR physiotherapy OR “physical therapy” OR “physical activity”).

The *inclusion criteria* were original studies published between January 2017 and January 2024 focusing on adult persons (≥ 18 years) with chronic low back pain who received exercise-based telerehabilitation delivered via any kind of technological device. The study should have been written in English and available in full text.

The *exclusion criteria* were conference papers, study protocols, case reports, studies describing a digital intervention delivered by virtual reality and serious gaming as well as those delivered only by telephone calls.

### Data management

All identified studies were independently and carefully reviewed by two authors [JS, KÅ] and were evaluated based on the inclusion and exclusion criteria and accuracy in relation to the purpose of the study. Duplicates were manually excluded during the review process. These were considered first at combined title and abstract level, and subsequently, at full-text level, with conflicts resolved by a third author [NS]. All authors agreed on the final inclusion list.

### Data extraction and analysis

A data extraction form was created in Microsoft Excel. Data extraction was performed by a combination of two authors; one author [JS] extracted data from all studies and another author [KÅ] checked the extraction for accuracy. Data were extracted on study characteristics (author, year, country, study design), population characteristics (health condition, sample size, gender, age) and intervention characteristics (type of exercise-based telerehabilitation intervention, additional intervention, group or individual intervention, technology used, follow-up, outcome, outcome measurements, key findings and future recommendations). According to the framework of Arksey and O’Malley, findings will be presented in narrative form [31].

## Results

### Study selection

**Fig. 1:**
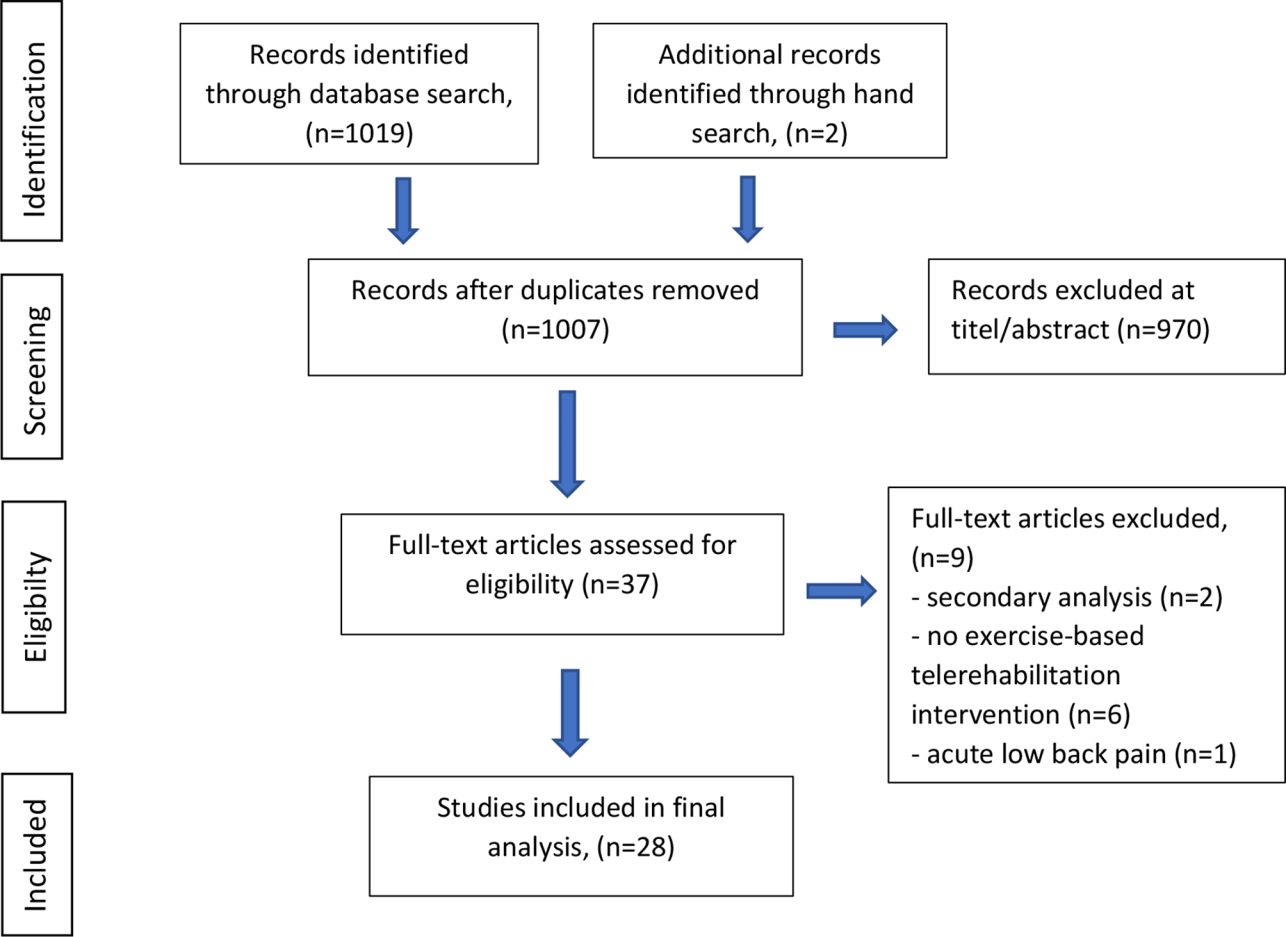
Flowchart

A total of 1019 articles were identified through database searches, and two additional articles were identified by manual search. After removal of duplicates, titles and abstracts were analysed based on the inclusion criteria and study purpose. A full-text screening of 37 articles was then performed, resulting in 28 studies that were included in the final analysis [35–62]. A PRISMA flowchart summarising the study selection process is shown in Fig. 1.

### Study characteristics

**Fig. 2:**
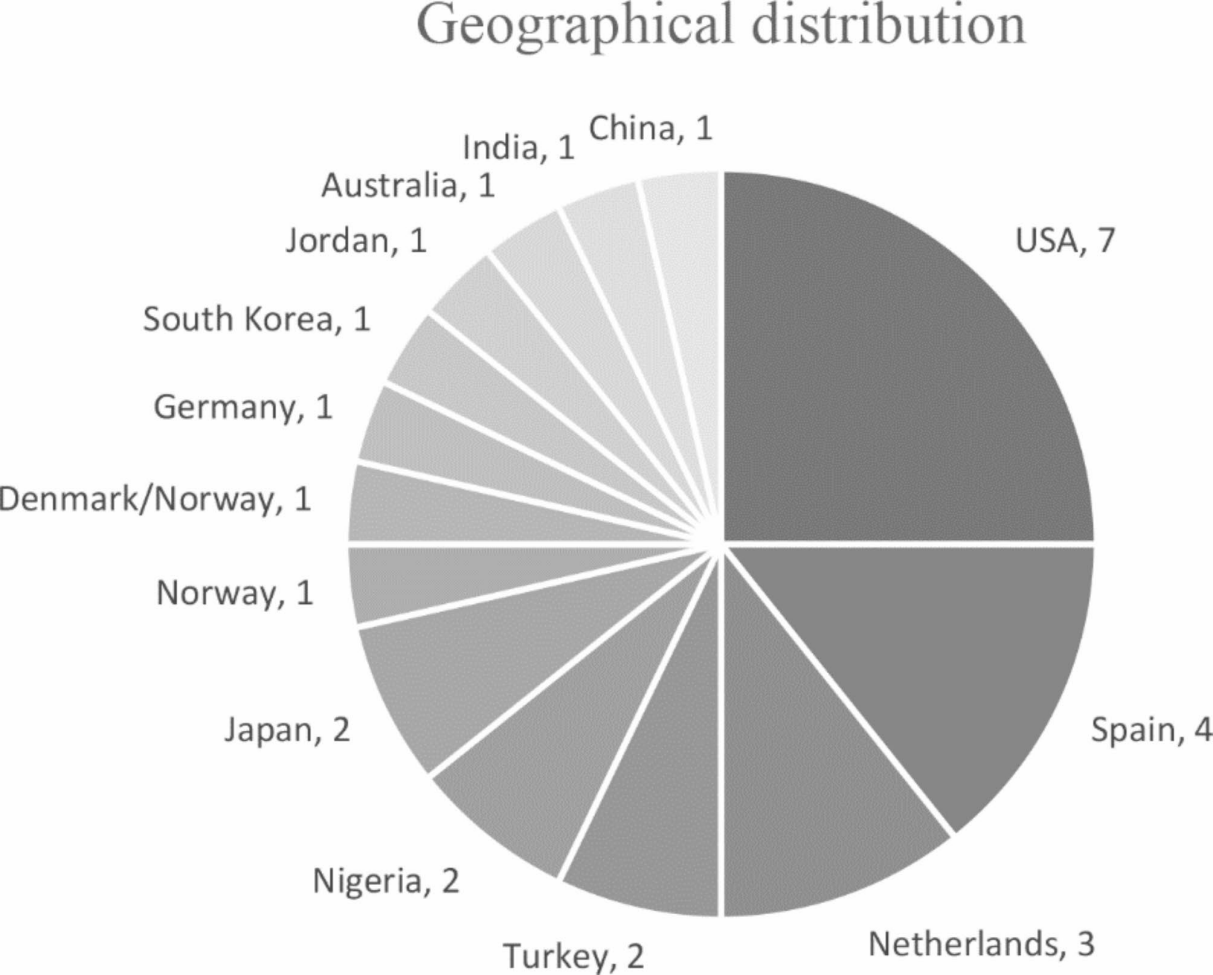
Geographical distribution of the included studies

**Fig. 3:**
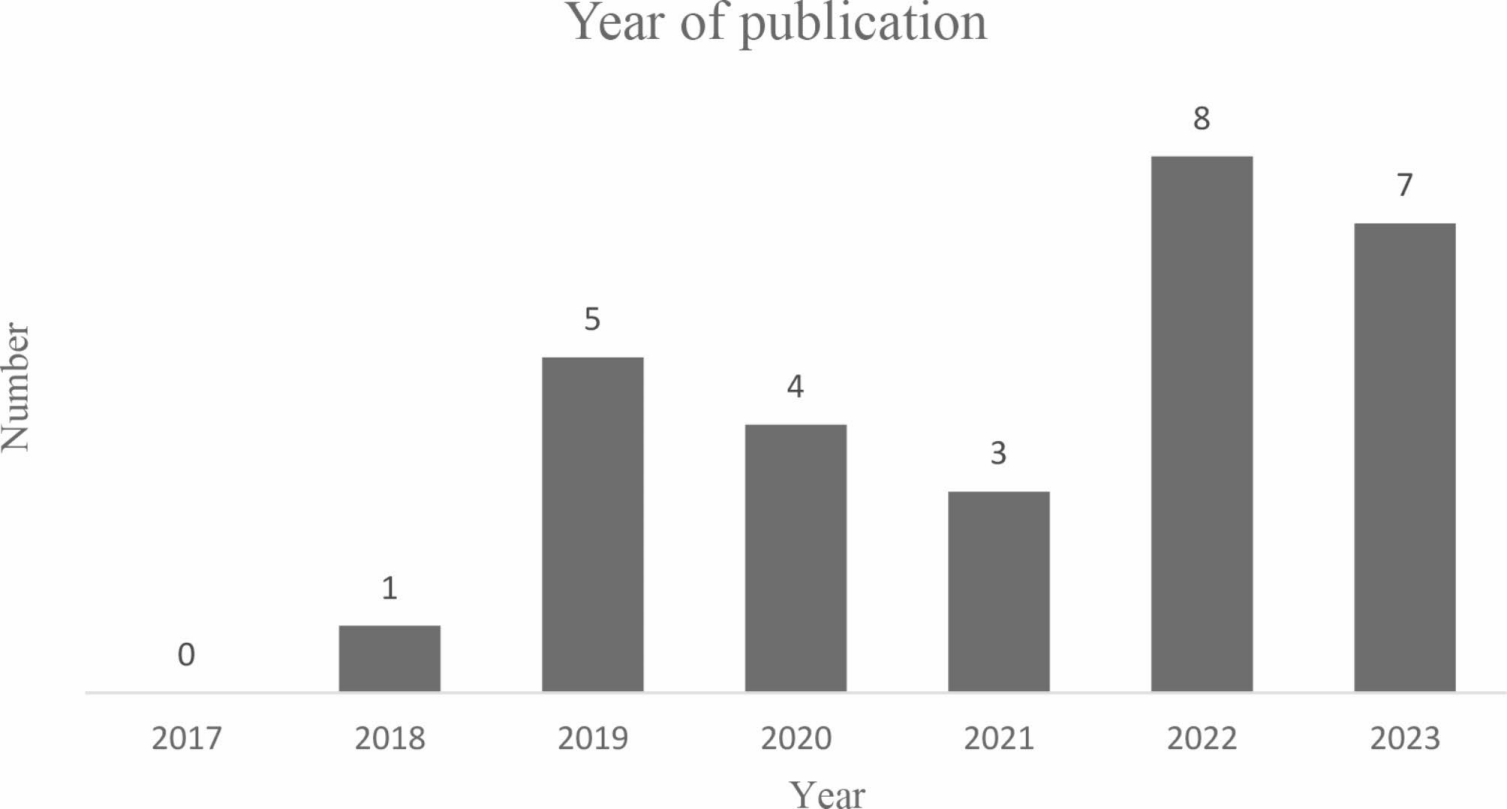
Year of publication

The included studies showed a wide geographical distribution, with studies conducted in 14 different countries. The United States was the most common (*n* = 7/28, 25%) followed by Spain (*n* = 4/28, 14%) and the Netherlands (*n* = 3/28, 11%), Fig. 2. Most studies were published in 2022 (*n* = 8/28, 29%) and 2023 (*n* = 7/28, 25%), Fig. 3. More than half of the included studies were randomised controlled trials (RCTs) (*n* = 15/28, 54%), but there were a variety of designs. Sample size ranged from 18 to 6468. Participants were aged 18 years and over, and both genders were represented in the studies. Characteristics of the included studies are presented in Table 1.

**Table 1:** Characteristics of the included studies

Author; Year; Country	Design	Sample size % women	Age	Technology	Telerehabilitation intervention	Group/ Individual	Control group	Outcome measures		Follow up duration
Almadawi et al.; 2020; Jordan	RCT	*N* = 41 54%	30–55	Smartphone Application Asynchronous	6w stretching, strength	Individual	Information	pain, disability, quality of life, sleep		6 w
Anan et al.; 2021; Japan	RCT	*N* = 94 23%	20–64	Smartphone Chat-bot Asynchronous	stretching, posture, mindfulness	Individual	Usual care, Regular exercise	pain		12 w
Bailey et al.; 2020; USA	Retrospective longitudinal study	*N* = 6468 50%	18–80	Tablet Application Asynchronous	12 w, 3/w stretching, strength, aerobic	Individual	No	pain, mental health, work productivity		6,12 w
Cheng et al.; 2022; China	RCT	*N* = 40 65%	18–65	Smartphone Application Asynchronous	6 w, 3/w stretching, strength	Individual	TR minus education	function, pain, depression, anxiety, quality of life		6,18 w
Chhabra et al.; 2018; India	RCT	*N* = 93 -	≥ 18	Smartphone Application Asynchronous	12 w, 1/day back and aerobic exercises	Individual	Conventional Physiotherapy	pain, disability/function, daily physical activity		12 w
Cui et al.; 2023; USA	RCT	*N* = 140 68%	18–80	Tablet App/Platform Asynchronous	8 w, 3/w exercises	Individual	Conventional Physiotherapy	function, pain, fear avoidance, mental health		4,8 w
Fatoy et al.; 2020; Nigeria	RCT	*N* = 47 -	20–65	Smartphone Application Asynchronous	McKenzie	Individual	Clinic based McKenzie	disability, quality of life, cost effectiveness		4,8 w
Fritz et al.; 2022; USA	Prospective longitudinal study	*N* = 126 63%	18–64	Smartphone Application/ Platform Synchronous videoconferencing	8 w, strength, conditioning, flexibility, motor control	Individual	No	disability/function, pain		10,26 w
Garreta-Catala et al.; 2023; Spain	RCT	*N* = 18 72%	18–67	Videoconferencing Synchronous	8 w, 1/w strength, resistance exercise.	Group	Standard care	feasibility, quality of life, disability, physical activity		2,6 m
Itoh et al.; 2022; Japan	RCT	*N* = 99 44%	20–64	Smartphone/Tablet Application Asynchronous	12 w, 1/day posture, mobility, strength, aerobic	Individual	Routine medical care	work productivity, pain, quality of life, fear of movement		4,8,12 w
**Author; Year;** **Country**	**Design**	**Sample size** **%women**	**Age**	**Technology**	**Telerehabilitation** **intervention**	**Group/** **Individual**	**Control group**	**Outcome measures**		**Follow up duration**
Karaduman et al.; 2023; Spain	RCT	*N* = 66 45%	≥ 18	App Video conferencing Synchronous	4 w, 3/w stabilization	Individual	Conventional Physiotherapy	function, pain, kinesiophobia		4 w
Kloek et al.; 2019; Netherlands	Feasibility study	*N* = 41 56%	18–65	Application Asynchronous	12 w, 3/w strength, mobility, relaxation	Individual	No	functional ability, pain, physical activity, sedentary behaviour, fear-avoidance		12 w
Koppenaal et al.; 2022; Netherlands	RCT	*N* = 208 49%	≥ 18	Smartphone Application Asynchronous	12 w tailored exercises	Individual	Conventional physiotherapy	disability/function, pain, fear avoidance, quality of life		12 w
Maracuzzi et al.; 2023; Norway	RCT	*N* = 294 59%	≥ 18	Smartphone Application Asynchronous	flexibility, strength, physical activity	Individual	Usual care	musculoskeletal health, disability, pain, self-efficacy, quality of life		6 w, 3,6 m
Mayer et al.; 2020; USA	RCT	*N* = 264 88%	27–44	Telehealth system Asynchronous	core, strength	Individual	Conventional Physiotherapy, Information		lost work time, core muscular endurance, function/disability, quality of life	3,6,9,12 m
Mbada et al.; 2019; Nigeria	Quasi experimental study	*N* = 47 72%	20–65	Smartphone Application Asynchronous	8 w, 3/w McKenzie	Individual	Clinic based McKenzie		pain, muscle endurance, disability/function	4,8 w
Park et al.; 2022; South Korea	Clinical trial	*N* = 54 78%	40–59, 65–79	Smartphone Web video program Asynchronous	8 w, 2/day flexibility, strength	Individual	No		pain, muscle strength, disability, quality of life	8 w
Raiszadeh et al.; 2021; USA	Prospective cohort study	*N* = 1090 59%	18–85	Platform Asynchronous	12 w core, strength	Individual	Conventional Physiotherapy		pain, disability, goal achievement, opioid use	12 w
Sandal et al.; 2021; Denmark/Norway	RCT	*N* = 461 55%	≥ 18	Smartphone Application Asynchronous	- physical activity, strength, flexibility	Individual	Usual care		disability, pain, ability to cope, fear avoidance, illness, quality of life	6 w, 3,6,9 m
Shebib et al.; 2019; USA	RCT	*N* = 177 41%	≥ 18	Tablet Application Asynchronous	aerobic activities	Individual	Education		pain, disability, surgery	4,8,11 w
**Author; Year;** **Country**	**Design**	**Sample size** **%women**	**Age**	**Technology**	**Telerehabilitation** **intervention**	**Group/** **Individual**	**Control group**		**Outcome measures**	**Follow up duration**
Stiges et al.; 2022; Spain	Clinical trial	*N* = 50 66%	18–59	Smartphone Application Asynchronous	4 w, 2/w strength, motor control, flexibility	Individual	Conventional physiotherapy		brain activity, pain, disability	4 w
Toelle et al.; 2019; Germany	RCT	*N* = 94 70%	18–65	Smartphone Application Asynchronous	strength, flexibility	Individual	Conventional Physiotherapy Information		pain, function, mental and physical aspects of chronic pain, quality of life	6,12 w
Vad et al.; 2022; USA	Clinical trial	*N* = 39 64%	18–65	Smartphone Application Asynchronous	3 m, 1/day flexibility, strength, core, aerobic	Individual	No		pain, function, pain medicine intake	3,6 w, 3 m
Villatoro-Luque et al.; 2023; Spain	RCT	*N* = 71 51%	18–65	Smartphone Platform Asynchronous	8 w, 2/w strength, mobilization	Individual	Conventional Physiotherapy		pain, disability, pain catastrophizing, strength	8 w, 3 m
Villatoro-Luque et al.; 2023; Spain	RCT	*N* = 68 50%	18–65	Smartphone App Videoconferencing Synchronous	8 w, 2/w exercises	Individual	Conventional Physiotherapy		pain, ROM, kinesiophobia	8 w, 3 m
Zadro et al.; 2019; Australia	RCT	*N* = 60 52%	≥ 55	Video game Wii Fit U software Asynchronous	8 w, 3/w flexibility, strength, aerobic	Individual	Usual activities		pain self-efficacy, care seeking, pain, disability/function, kinesiophobia	8 w, 3,6 m
Özden et al.; 2022; Turkey	RCT	*N* = 50 60%	18–65	Platform Asynchronous	8 w, 1/day strength, stretching, mobility	Individual	Conventional physiotherapy		pain, disability/function, quality of life, kinesiophobia	8 w

RCT, randomized controlled trial; TR, telerehabilitation, w,weeks, m, months

### Technology and exercise intervention

The included studies described different technological solutions, devices and modes of delivery to convey the telerehabilitation. The studies showed a variation in technological method used. Smartphone was the most used digital device (*n* = 16/28, 57%), followed by tablet (*n* = 5/28, 18%) and video game equipment (*n* = 1/28, 4%). The exercise intervention, which typically consisted of different types of exercise therapy, where strengthening was most common followed by mobility, stabilisation and aerobic exercise, usually in combination and delivered asynchronously (*n* = 25/28, 89%) via an application (*n* = 17/28, 61%). There were often some educational and motivational elements embedded in the telerehabilitation, where the applications provided the participants with instructional exercise videos and messages to remind them to do the exercises, as well as educational and motivational notifications. Four studies [40, 41, 45, 62] were based on an application which used patient-related information (i.e., symptoms, completion of exercises, etc.) collected earlier to offer individually tailored recommendations. Other technological solutions used in the studies were web-based platforms (*n* = 4/28, 14%), chat bot (*n* = 1/28, 4%) and Wii Fit U software (*n* = 1/28, 4%).

Four studies [35, 57, 59, 60] used real-time videoconferencing to convey exercise instructions and to offer information and follow-up. The exercises, though, were usually carried out with the support of pre-recorded video modules. The study by Gerrata-Catala was the only one evaluating a group exercise intervention and thus also the only one to perform a group exercise programme via videoconferencing.

All included studies described a wide variation in the duration of interventions (range 4 weeks to 12 months), the frequency of the exercise programme (range 2 times per day to 1 time per week) as well as different intervals for follow-up (range 4 weeks to 12 months). For detailed information of technology and exercise intervention, see Table 1.

### Outcome measures and clinical findings

The included studies showed a large variation regarding outcome measures. The most commonly evaluated clinical outcome measure was pain (*n* = 25/28, 89%), followed by disability/physical function (*n* = 23/28, 82%), quality of life (*n* = 12/28, 43%) and kinesiophobia/fear avoidance (*n* = 10/28, 36%). All studies used mainly self-report questionnaires or scales to evaluate clinical outcomes.

In the studies evaluating pain, the numerical rating scale (NRS) (*n* = 14/25, 56%) and visual analogue scale (VAS) (*n* = 9/25, 36%) were the most common measurements used. Regarding disability or physical function, the Oswestry Disability Index (ODI) (*n* = 17/23, 74%) and Roland Morris Disability Questionnaire (RMDQ) (*n* = 6/23, 26%) were used. Quality of life was evaluated by using the questionnaires EQ-5D-5 L (*n* = 4/12, 33%), SF-36 (*n* = 4/12, 33%), SF-12 (*n* = 3/12, 25%) and SF-6D (*n* = 1/12, 8%). Kinesiophobia and fear avoidance was most commonly evaluated using the Tampa Scale of Kinesiophobia (TSK) (*n* = 6/10, 60%) and Fear Avoidance Beliefs Questionnaire (FABQ) (*n* = 4/10, 40%).

Half of the included studies compared exercise-based telerehabilitation with conventional face-to-face exercise therapy (*n* = 14/28, 50%), while other studies compared telerehabilitation with information, education, routine medical care, or usual activities (*n* = 9/28, 32%). When telerehabilitation was compared to conventional face-to-face exercise therapy, no statistically significant difference between groups were seen. Both groups showed similar clinical improvements in pain and disability/physical function [38, 40, 41, 47, 51, 53, 56–59, 61]. However, the studies by Özden [36], Chhabra [49] and Toelle [54] reported significant improvements in pain and/or improvements in disability/physical function in patients randomised to telerehabilitation compared to a conventional exercise intervention.

Telerehabilitation showed significant improvements in pain and disability/physical function in most (*n* = 7/9, 78%) of the studies analysed [39, 43, 44, 46, 52, 55, 60] compared to interventions which involved only information, education, routine medical care and/or usual activities. However, a few studies (*n* = 2/9, 22%) [50, 62] reported no significant difference between groups in pain and/or disability/physical function. All studies without a control group (*n* = 5/28, 18%) [35, 37, 42, 45, 48] showed statistically significant clinical improvements in pain and disability/physical function after the telerehabilitation intervention.

## Discussion

The studies identified in this scoping review showed a great variability regarding origin, which shows that many countries around the world are working to integrate digital alternatives in healthcare. It is also clear that this research area is rapidly growing, with 54% of the included studies published the last two years. This may be a probable consequence of the Covid-19 pandemic, when conventional physiotherapy was put on hold or converted to digital solutions. But there is also a growing interest in sustainable, evidence-based, digital alternatives, which may be resource effective as more people are getting old.

The included studies described a variation in technological methods used. The exercise instructions were most often delivered asynchronously via an application using a smartphone. These findings are in line with the previous reviews of Lara-Palomo [25] and Tabacof [63], who both described the asynchronous mode of delivery via an application as the most frequently used. In contrast, Jirasakulsuk [24] described real-time, synchronous, telerehabilitation via a computer platform. This difference could be due to the inclusion of older adults, aged 60 years and over, who may prefer computers and supervised interventions.

The exercise interventions in the included studies were heterogeneous in terms of the content of the exercise therapy, duration of intervention, frequency of exercise programme and follow-up, which is in line with previous reviews [25, 26, 63]. However, most studies included both training and information, which follows the recommendation according to guidelines [7]. Few studies conducted long-term follow-up. Only 6 (of 28) studies had a follow-up period of 6 months or longer, and only one study had 12 months’ follow-up, which leaves a knowledge gap regarding the long-term effects of telerehabilitation in chronic low back pain.

There are previous reviews evaluating effectiveness of telerehabilitation from different perspectives, such as within the context of physiotherapy [26, 64], for various health conditions such as musculoskeletal disorders [22–24, 65, 66] and some reviews evaluating solely the effect of telerehabilitation in patients with low back pain [25, 63, 67]. However, there has previously been a lack of a review that map different types of exercise-based telerehabilitation for patients with chronic low back pain.

In the present review of studies evaluating telerehabilitation compared with conventional face-to-face exercise, both groups showed significant clinical improvements, and there was no difference between groups. Studies evaluating telerehabilitation compared to a control group with content other than conventional exercise, or no control group at all, showed significant clinical improvements after the telerehabilitation intervention. These results align with the results of previous studies. Lara-Palomo [25] concluded that eHealth interventions are as effective regarding: pain and functional status as other face-to-face or home-based interventions. Chen [67] and Cotrell [22] showed that telerehabilitation in addition to usual care was more favourable than usual care alone. Furthermore, Cotrell also showed that telerehabilitation solely was equivalent to face-to-face intervention. However, previous research proves contradictory: Vieira [66] reported that two of three high quality systematic reviews lacks evidence for nonspecific low back pain. In summary, the results of previous research are inconsistent, both supporting and questioning the effectiveness of telerehabilitation.

Furthermore, to develop sustainable digital solutions to convey exercise-based telerehabilitation, it is important to consider whether any added value emerges from the new technology. Then, the SAMR model, with its four hierarchical levels can serve as a guide. The digital solutions described in the present review have certain functional improvements, such as the patient being able to access the exercise programme via an application or QR code and thus see the exercises as a video, which is intended to provide for better learning. Another functional improvement is the use of a chat feature making it possible to interact. These characteristics are examples of augmentations compared to conventional physiotherapy, where home-based exercise programmes are mainly used. At the modification level, the new technology allows for a task redesign, and a shared contact surface is used. Synchronous, real-time videoconferencing with supervised exercise, individually or in group, are examples of such modifications described in the present review. This technological development with the possibility of real-time dialogue and feedback from the physiotherapist contributes to the correct execution of the exercises and positively impacts the patients’ outcomes. In addition, exercise therapy through real-time videoconferencing contributes to increased accessibility and sustainability, by saving time and travelling for patients, compared to conventional face-to-face physiotherapy.

There is a growing interest in digital solutions and the field of telerehabilitation for chronic low back pain is heterogeneous and the interventions are diverse. The design of a scoping review was therefore suitable to answer the purpose of how telerehabilitation is used and to map existing research. The design thus involves the inclusion of studies with different study designs and heterogeneous content [32]. In fact, the included studies showed a great variability in terminology regarding the telerehabilitation, with various types of exercise therapy interventions and different technological solutions. Furthermore, the sample size varied widely, as did the type of outcome measurements and follow-up period, which makes comparison of the included studies difficult. Most of the included studies also describe a combination of different types of exercise therapy in the intervention, which makes it difficult to evaluate the effect of each specific exercise type. A risk-of-bias assessment to evaluate study quality was not performed, as is typical in scoping review methodology [31].

### Limitations and strengths

To our knowledge, this is the first scoping review focusing on mapping and evaluating types of exercise-based telerehabilitation used in chronic low back pain. This review has clearly mapped the field and highlighted digital solutions that provide added value for the patient and the healthcare. The strengths of this study relate to the use of a methodological framework for scoping reviews [31] and the clarity and transparency by following a pre-registered study protocol. The results, though, also need to be considered in relation to some possible limitations. Some relevant studies may have been missed due to the selection criteria; the databases used for the search, including the search strings; and the inclusion only of full text studies published in English. Another possible limitation was the exclusion of digital interventions delivered by virtual reality and serious gaming. This delimitation was based on our focus on exercise therapy and not gaming. However, both virtual reality and serious gaming are increasing areas within telerehabilitation and may therefore also be interesting to evaluate in future research.

The included studies agree that further research on exercise-based telerehabilitation is needed, including more robust study designs, larger sample sizes and long-term effects. As there was only one identified study evaluating a digital group exercise intervention and only one using synchronous, real-time videoconferencing for the exercise therapy sessions, the present review has demonstrated some knowledge gaps in the literature. Questions interesting for future research regard cost-effectiveness and development of sustainable and resource-effective healthcare, and further, the identification of which patients are most likely to respond to telerehabilitation and which will not. Research should also evaluate how telerehabilitation and conventional physiotherapy can most suitably be combined.

## Conclusions

This scoping review confirms the heterogeneity within this rapidly growing research area, but also contributes by mapping and summarising key concepts and identifying knowledge gaps and the need of further research. Exercise-based telerehabilitation is most typically delivered asynchronously with instructional exercise videos via a smartphone application. The digital solutions described in this review provide added value through functional improvements as well as task redesign for the benefit of patients and a more accessible and sustainable healthcare.

## Data Availability

The dataset used and analysed during the current study are available from the corresponding author on reasonable request.

## Abbreviations

SAMR: The Substitution, Augmentation, Modification, and Redefinition framework
PCC: Population, Concept and Context
OSF: Open Science Framework
RCT: Randomised Controlled Trial
NRS: Numerical rating scale
VAS: Visual analogue scale
ODI: Oswestry disability index
RMDQ: Roland Morris disability questionnaire
TSK: Tampa scale of kinesiophobia
FABQ: Fear avoidance beliefs questionnaire
